# Some New Methods of Radiotherapy

**Published:** 1954-10

**Authors:** R. C. Tudway

**Affiliations:** Bristol Royal Hospitals


### SOME NEW METHODS OF RADIOTHERAPY

BY

R. C. TUDWAY, M.B., B.S., B.SC., F.F.R.
*Bristol Royal Hospitals*

There have been many advances in treatment with X-Rays and radio-active substances. This is an account of those recently adopted and those to be used in future in the Radiotherapy Department at Bristol.

There are two great difficulties in using Deep X-Rays for the treatment of malignant disease. First, " Deep " X-Rays do not penetrate really deeply. Their effective range is a few centimetres deeper than the skin, and it is still impossible with ordinary Deep X-Rays to give an adequate dose to the centre of the pelvis, to lung roots and oesophagus, or even to deeper lymph glands in the supraclavicular region. This is possible, however, by using the X-Rays generated by voltages between two and ten million volts. These rays and machines are called " supervoltage ".

The second difficulty under which the X-Ray therapist labours is that the dose of rays needed to get rid of a growth is commonly rather close to the dose which will excessively damage the normal tissue within the beam. In other words the " therapeutic ratio " is poor. Here also supervoltage rays have a definite advantage because bone and cartilage, which are very easily damaged by ordinary X-Rays, are not so easily damaged by supervoltage X-Rays. In fact all the unwanted reactions of the patient, both skin effects and radiation sickness, are definitely less with supervoltage. Supervoltage machines are, however, very large and costly—for example, a four-million-volt machine ( a " linear accelerator ") costs about £40,000.

### RADIO-ACTIVE COBALT

Since the atomic energy piles at Harwell and elsewhere have been working, it has been possible to make radio-active cobalt. Radio-active cobalt has one useful property; it emits rays nearly the same as those from a three-million-volt X-Ray machine. This means that if we can get enough radio-active cobalt and enclose it in the middle of a large mass of protecting material, we can get a beam of 2–3 million volt rays simply by leaving a hole in the side of the protecting mass.

Up till now only Canada and the U.S.A. have been able to provide enough radio-active cobalt to give as much power as an actual supervoltage machine. Such a cobalt beam unit from Canada has been presented to Mount Vernon Hospital, Middlesex, and has [illegible]een installed there.

We [illegible] Bristol are now installing a cobalt beam unit, big enough to compete with super[illegible]age X-Ray machines; it is the first to be made entirely in Great Britain. It should be ready in July this year. At first our unit will not be quite up to the American strength because this country cannot yet provide such a large supply of cobalt but we are expecting that we may have more cobalt later on, and our machine is designed to hold the full amount of 1,500 curies. It is in some ways a handier machine to use as well as being cheaper to maintain, and, in my opinion, it will meet a widely felt need.

Patients with carcinoma within the true pelvis, some with carcinoma of the breast and carcinoma in the head and neck, will be those most likely to benefit from this new machine.

Frequently, after operation for carcinoma of the cervix uteri or after the use of radium in this disease, Deep X-Rays are necessary to treat the side walls of the pelvis to destroy any deposits left there. This has never been easy to do with ordinary Deep X-Rays. The cobalt beam will not only give a much better dose to these parts but will cause less damage to the skin, bone will no longer be an obstacle, and the patient will have less constitutional upset. We can now give a dose which may destroy a majority of these deposits of growth instead of only a few of those which are very radio-sensitive.

The amount of Deep X-Rays for carcinoma around the larynx is usually limited by the fact that perichondritis is caused by a dose lower than that which will burn soft tissues. Carcinoma of the larynx must therefore be given a lower dose of Deep X-Rays than the same type of growth in other sites. Cartilage, however, has little more sensitivity to rays from the cobalt unit, so with this unit a better dose can be given to the larynx. Most growths of the jaws and sinuses involve bone or are surrounded by bone. These also are better treated by the cobalt beam because bone is another tissue which is selectively damaged by Deep X-Rays and is an obstacle to them; but the cobalt beam treats bone in the same way as soft tissue, and therefore has none of these disadvantages. Likewise the bone of the skull will not absorb rays from the cobalt unit intended for intracranial tumours as it would absorb Deep X-Rays.

We have had to face some difficulties in the construction of the machine, and have now been working on the project for two years. The cobalt is very radio-active indeed, and must lie in the centre of a cylinder of lead two feet in diameter, otherwise the whole of the patient and the staff of the department would be irradiated. No individual can approach within a yard of the unprotected source for more than a few seconds without receiving a dose which could be harmful. A two-foot-diameter mass of lead is very heavy and unwieldy, so we are using uranium metal instead of lead. The uranium cylinder need only be eleven inches in diameter.

One advantage of uranium is that the units can be supported on commercially built stands like those used for Deep X-Ray machines, because they only weigh 600 pounds each, whereas the lead protected unit weighs over 2,000 pounds and must have a specially built support. The units are raised, lowered and rotated for the aiming of the beams by means of electric motors in the supporting stands.

When the shutter is open and the beam is in use, it is essential to prevent rays from escaping from the room. This means that the walls of the room must be of a thickness varying from eighteen inches to three feet. This has presented a major problem in the Bristol General Hospital where there is no room to build anew, and such heavy new walls could only be supported at ground level. A solution has been found in the arched cellars which support the internal courtyard of the building. These arches have walls about two and a half feet thick which form an excellent protective surround to the treatment rooms. As can well be imagined these cellars in their crude state are not very inviting for the treatment of patients. The major work of the whole project is the heating, ventilation, lighting and decorating of these rooms. Doors to prevent escape of rays would have to be very heavy—equivalent to concrete eighteen inches thick. Fortunately the rays have very little ability to turn corners so their exit from the rooms can be blocked by having a twisting passage with thick walls for the entrance. The provision of such tortuous entrances to the rooms has meant careful planning to fit them within the existing walls. In other places doors like those of ordinary X-Ray rooms are also being used. The electrical controls can then be so arranged that anyone who accidentally enters the room while the unit is in action will automatically turn it off.

The cobalt unit which is being installed at the Bristol General Hospital is a double unit, with two separate parts in two adjacent rooms so that two patients will be treated at the same time. This has been done to minimize the main disadvantage of radio-active cobalt which is that the radio-activity gradually fades away and after five years only half the strength is left. This means that after about four years it will be too weak to treat the deepest tumours and must be renewed. The cobalt in one treatment room will be used for these deep tumours for four years. Then it will be transferred to the other room for a further four years where it will be used for the treatment of more accessible tumours which are sensitive to lower doses.

Radio-active cobalt can also be used to replace radium in needles. It has the advantage that, unlike radium, it does not need to be kept in a platinum needle. The cobalt needles can be made of stainless steel which is much stronger than platinum. We have made them at the Bristol General Hospital, and we find them very satisfactory. The action of these needles on the patient and on the growth is indistinguishable from that of radium. Loss of a cobalt needle is less serious than that of radium.

### RADIO-ACTIVE CAESIUM

Before long, radio-active caesium should be available. This substance is one of the waste products from the atomic piles. It cannot be used in an impure state, but if a purification plant is set up it can be made available in large quantities. It emits rays which are not quite so powerful as those from cobalt; they are nearly equal to those from a one-million-volt X-Ray machine.

### RADIO-ACTIVE IRIDIUM

We are also using radio-active iridium in carcinoma of the cervix uteri. The reason for this is that the uterus and vagina tolerate radium extremely well and can take a dose heavy enough to reach the lymph glands of the side wall of the pelvis. Unfortunately the bladder and rectum are much more sensitive structures. Attempts have been made to introduce screens of platinum between the radium and the rectum, but unfortunately the rays from radium are so penetrating that very heavy and bulky screens must be used. The rays of radio-active iridium penetrate the tissues easily, but are still not too energetic to be stopped by a small screen, especially if the latter is made of uranium.

### SYSTEMIC USE OF RADIO-ACTIVE ISOTOPES

In the last year or two, there have been no great advances in the systemic use of radio-active isotopes; but methods have been improved.

The use of radio-active iodine in thyroid disease is now well established. Carcinoma of the thyroid may sometimes take up radio-active iodine and enough may be concentrated to destroy the growth. Unfortunately the normal thyroid tissue tends to absorb the greater part of the administered iodine; so the first step in this treatment is usually to remove the normal thyroid. Occasionally the tumour tissue absorbs iodine so actively that this step is not necessary. We have successfully treated one such case. This patient's growth had recurred after both surgery and Deep X-Rays. A single large dose of radio-active iodine was then given, the swelling disappeared and she has now been free of recurrence for two years.

### THYROTOXICOSIS

Radio-active iodine is of value in those cases of toxicosis in which surgery is unsafe, and in which the thiouracil group of drugs is not tolerated or has lost effect. In primary Graves' disease it is usually possible to achieve a euthyroid state with radio-active iodine, though there is always some risk of leaving the patient somewhat myxoedematous. In secondary thyrotoxicosis from nodular goitres the correct dosage is very difficult to judge and over-treatment with consequent myxoedema is quite common. However, this may be helpful because the patients with secondary thyrotoxicosis often have some heart failure, and the sub-thyroid state is an advantage. In our experience the treatment of these secondary toxic goitres with radio-active iodine has been very successful. So far we have treated about a hundred patients and our results will soon be published.

### HEART DISEASE

In some cases of heart disease not due to thyrotoxicosis, radio-active iodine is given deliberately to produce some myxoedema and so relieve the work of the heart. The iodine is given by mouth. It produces no reactions apart from slight nausea.

### RADIO-ACTIVE PHOSPHORUS

Systemic radio-active phosphorus has been tried for the treatment of malignant disease, and rapidly growing tissue, such as a tumour, will take up more phosphorus than normal tissues. The results however are not good. Phosphorus is stored in bone; therefore tumours originating within a bone may be treated if they are sufficiently sensitive. Both myeloid and lymphatic leukaemia respond well for a while. Other reticuloses, of which mycosis fungoides is one, sometimes respond well. In multiple myelomatosis there may be considerable, though temporary, relief.

### FUTURE RESEARCH

Little is known about what makes a tumour sensitive to radiation. At the moment various lines of research are in hand which include increasing oxygen uptake by a tumour during radiotherapy, and administration of vitamin K. Neither approach has proved promising so far.

Other work is already needed to help us select those patients most likely to benefit from radiotherapy, for histological appearances are a poor guide and we have often to resort to trial and error.

Progress in radiotherapy is rapid and before long we may have the answers to some of these outstanding problems.

